# Coronary atherosclerotic burden in patients with embolic stroke of undetermined source

**DOI:** 10.1093/esj/23969873251381912

**Published:** 2026-01-01

**Authors:** Yaron Aviv, Rani Barnea, Chen Gurevitz, Lior Fuchs, Gideon Shafir, Eitan Auriel, Mark Kheifets, Ran Kornowski, Ashraf Hamdan, Inbar Nardi Agmon

**Affiliations:** Division of Cardiology, Rabin Medical Center, Petah Tikva, Israel; Gray School of Medicine, Tel Aviv University, Tel Aviv, Israel; Gray School of Medicine, Tel Aviv University, Tel Aviv, Israel; Department of Neurology, Rabin Medical Center, Petah Tikva, Israel; Division of Cardiology, Rabin Medical Center, Petah Tikva, Israel; Gray School of Medicine, Tel Aviv University, Tel Aviv, Israel; Gray School of Medicine, Tel Aviv University, Tel Aviv, Israel; Gray School of Medicine, Tel Aviv University, Tel Aviv, Israel; Department of Radiology, Rabin Medical Center, Petah Tikva, Israel; Gray School of Medicine, Tel Aviv University, Tel Aviv, Israel; Department of Neurology, Rabin Medical Center, Petah Tikva, Israel; Division of Cardiology, Rabin Medical Center, Petah Tikva, Israel; Gray School of Medicine, Tel Aviv University, Tel Aviv, Israel; Gray School of Medicine, Tel Aviv University, Tel Aviv, Israel; Division of Cardiology, Rabin Medical Center, Petah Tikva, Israel; Gray School of Medicine, Tel Aviv University, Tel Aviv, Israel; Division of Cardiology, Rabin Medical Center, Petah Tikva, Israel; Gray School of Medicine, Tel Aviv University, Tel Aviv, Israel

**Keywords:** Embolic stroke of undetermined source, calcium score, atherosclerosis

## Abstract

**Introduction:**

Embolic Stroke of Undetermined Source (ESUS) is a subtype of cryptogenic stroke with no clear etiology despite thorough evaluation. Atrial fibrillation (AF) is detected in only ~40% of cases, and trials of empiric anticoagulation have failed to reduce recurrence, suggesting other mechanisms such as subclinical atherosclerosis may contribute. Coronary artery calcium (CAC) scoring is a validated marker of atherosclerosis, yet its burden in ESUS remains underexplored.

**Patients and methods:**

We conducted a retrospective cohort study of consecutive ESUS patients admitted between April 2019 and December 2023 who underwent cardiac CT angiography (CCTA) during diagnostic work-up. CAC scores were calculated using the Agatston method, and percentiles were derived from the MESA database, adjusted for age, sex, and ethnicity. Patients with prior coronary interventions were excluded.

**Results:**

Among 165 ESUS patients (median age 73.0 [IQR 66.5–82.0]; 47.9% female), the median CAC score was 225 [IQR 41.5–623.5] AU, and the median CAC percentile was 65 [IQR 40.05–85.0], significantly higher than population norms (*p* < 0.001). Patients ⩽65 years had higher CAC percentiles than older patients (80.0 [58.2–90.7] vs 61.0 [36.0–80.0], *p* = 0.002), despite similar CAC scores (*p* = 0.396).

**Conclusion:**

ESUS patients exhibit a high burden of coronary atherosclerosis, particularly notable in younger individuals. Elevated CAC may reflect both subclinical atherosclerosis and a broader cardiovascular risk profile, offering insight into stroke pathophysiology and the limited efficacy of empiric anticoagulation. CAC assessment could improve etiologic classification and inform tailored secondary prevention.

## Graphical Abstract

**Graphical Abstract:**
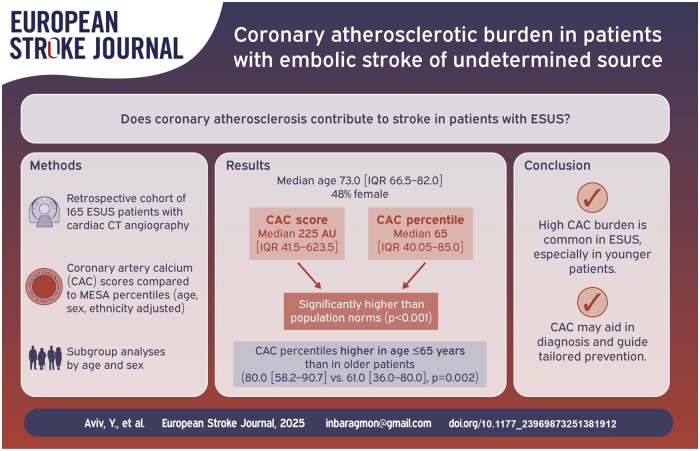

## Background

Embolic Stroke of Undetermined Source (ESUS) is a subset of cryptogenic stroke, defined as a non-lacunar infarct without significant arterial stenosis, major risk cardioembolic source, or other identifiable cause.^1,2^ Despite comprehensive diagnostic evaluation, the underlying mechanism remains elusive in many patients. Although atrial fibrillation (AF) is often suspected, it is detected in only ~40% of ESUS cases, even with prolonged cardiac monitoring.^3^ Randomized trials of empiric oral anticoagulants (OACs) in unselected ESUS population have failed to reduce stroke recurrence,^4–7^ suggesting that mechanisms beyond covert AF- such as non-stenotic atherosclerosis- may be contributory.^8^

Cardiac computed tomography angiography (CCTA) provides comprehensive imaging of both intra-cardiac and aortic embolic source.^9–11^ We previously demonstrated that gated CCTA identified intra-cardiac thrombi in 17% of ESUS patients^12^ and revealed an association between ESUS and complex left atrial appendage (LAA) morphology,^13^ highlighting its potential role in refining stroke mechanisms and guiding secondary prevention.

Coronary artery calcium (CAC) scoring is a validated maker of atherosclerosis, strongly associated with coronary events,^14^ ischemic stroke,^15^ and all-cause mortality.^16^ The risk of vascular events rises with increasing calcium burden.^17^ However, the prevalence and degree of coronary atherosclerosis in ESUS patients have not been systematically studied. This study aimed to assess CAC burden in a contemporary ESUS cohort and to compare findings to population-based reference percentiles to explore its potential mechanistic implications.

## Methods

We conducted a retrospective cohort study of consecutive patients admitted with ESUS to the stroke unit or neurology department at Beilinson Hospital, Israel, between April 2019 and December 2023. Patients were identified through the institutional medical records registrar based on a discharge diagnosis of ischemic stroke (IS); those with transient ischemic attacks (TIA) were excluded. All imaging and clinical data were reviewed by a stroke neurologist to confirm ESUS diagnosis, based on criteria aligned with major ESUS trials.^2^ Patients meeting these criteria were included in a dedicated ESUS registry.^12,13^ Within this registry, 226 patients underwent CCTA as part of their diagnostic workup were eligible for inclusion. Medical records of ESUS patients who did not undergo CCTA were also reviewed to document clinical reasons for alternative imaging, such as preference for transesophageal echocardiography (TEE) and patient factors such as age, renal function, ability to collaborate with breath holding techniques during the exam, or availability.

CCTA was typically performed during the index hospitalization, most often within the first 3–7 days after stroke onset, depending on patient stability and scanner availability. All CCTA scans were performed on Siemens Somatom Force or Philips Brilliance 256 scanners. Standardized acquisition protocols were used, including pre-scan beta-blockade (intravenous metoprolol up to 20 mg) for heart rates >65 bpm, administration of 55–85 mL of Iopromide 370 contrast at 4–6 mL/s followed by a 40 mL saline flush, and ECG-gated imaging during inspiratory breath-hold. The protocol included three acquisitions: (1) a non-contrast scan for CAC scoring, (2) an early contrast-enhanced angiographic scan using bolus tracking, and (3) delayed enhancement imaging initiated approximately 80–90 s after contrast administration.

Patients with prior coronary stenting or coronary artery bypass grafting (CABG) were excluded from the CAC analysis due to limited accuracy in quantification of calcium in the presence of metallic implants and altered native vessel morphology.^18^ CAC scoring was performed using Philips IntelliSpace v12.0 software by an expert cardiac imaging cardiologist blinded to clinical variables. A single reader was used, given low interobserver variability.^19^ Scores were reported in Agatston units (AU), and CAC percentiles were calculated using the MESA (Multi-Ethnic Study of Atherosclerosis) reference database^20^: this is a large population-based cohort used to establish normative CAC percentiles stratified by age, sex, and ethnicity. It provides reference percentiles that allow contextualization of individual CAC scores relative to a healthy population without clinical cardiovascular disease. CAC scores were categorized using standard thresholds: 0 (no calcification), 1–99 AU (mild), 100–399 AU (moderate), and ⩾400 AU (severe). These categories were used for descriptive purposes and figure presentation. However, statistical analyses—including subgroup comparisons—were conducted using CAC score and percentile as continuous variables, due to the highly skewed distribution. Percentile estimation was capped at age 85.

Statistical analysis was performed using SPSS v29. Continuous variables were expressed as mean ± standard deviation (SD) or median [interquartile range, IQR] as appropriate. The distribution of CAC scores was highly skewed, and therefore all CAC-related variables (score and percentiles) were analyzed using non-parametric methods. One-sample Wilcoxon signed-rank tests were used to compare CAC percentiles to the expected population median of 50%. Subgroup comparisons (e.g. by age or sex) employed chi-square, Wilcoxon rank-sum, or Mann–Whitney U tests, as appropriate. A two-tailed *p*-value < 0.05 was considered statistically significant.

## Results

A total of 226 ESUS patients underwent CCTA during the study period. After excluding 61 patients with prior coronary stenting or CABG, 165 patients were included in the CAC analysis cohort. The baseline characteristics of this population are shown in Table 1.

**Table 1.** Patient characteristics.

Entire cohort	*N* = 165	Groups according to CAC grade			
		Zero 0 AU *n* = 15	Mild 1–99 AU *n* = 37	Moderate 100–399 AU *n* = 57	Severe ⩾400 AU *n* = 56
Age, years	73.0 [66.5–82.0]	72.0 [66.0–73.0]	73.0 [64.5–79.0]	73.0 [64.5–80.5]	74.5 [69.2–85.0]
Female sex	79 (48)	11 (73)	17 (46)	30 (53)	20 (36)
BMI, kg/m^2^	26.6 [24.1–31.0]	27.9 [25.5–32.4]	26.7 [23.6–30.7]	25.5 [23.9–30.3]	26.9 [24.6–31.0]
Smoking status					
Former	28 (17)	1 (7)	7 (19)	14 (25)	6 (15)
Active	19 (11.5)	0 (0)	4 (11)	5 (9)	10 (18)
Comorbidities					
Hypertension	110 (67)	9 (60)	25 (68)	41 (72)	36 (62)
Diabetes mellitus	56 (34)	3 (20)	9 (24)	16 (28)	28 (50)
Coronary artery disease	19 (11.5)	0	1 (3)	4 (7)	11 (20)
Heart failure	10 (6)	0	0	2 (3.5)	8 (14)
Previous stroke	39 (24)	2 (13)	7 (19)	17 (30)	15 (27)
Peripheral artery disease	9 (5.5)	0	2 (5)	1 (2)	6 (11)
Chronic kidney disease	11 (7)	0	2 (5)	2 (3.5)	7 (12.5)
Ejection fraction	60.0 [60.0–65.0]	60.0 [60.0–65.0]	60.0 [60.0–65.0]	60.0 [60.0–65.0]	60.0 [50.0–60.0]
LDL cholesterol, mg/dL	100.0 [70.0–124.0]	91.0 [70.5–138.5]	111.0 [68.0–129.0]	102.0 [71.0–124.5]	98.0 [69.0–120.0]
Atherosclerotic changes					
Aortic arch	84 (51)	3 (20)	14 (38)	23 (40)	44 (78)
Carotid arteries	69 (42)	2 (13)	9 (24)	16 (28)	42 (75)

BMI: body mass index; LDL: low density lipoprotein.

Continuous variables are presented as median [interquartile range]. Categorical variables are presented as *N* (%).

The median age was 73.0 [interquartile range (IQR) 66.5–82.0] years, and 48% were female. As to cardiovascular risk factors, 28.5% were ever smokers, 67% had hypertension, 34 had diabetes mellitus and the median low-density lipoprotein (LDL) cholesterol was 100.0 [IQR 70.0–124.0] mg/dL. A history of prior stroke was present in 24%, and 11.5% had prior myocardial infarction.

**Figure 1.**
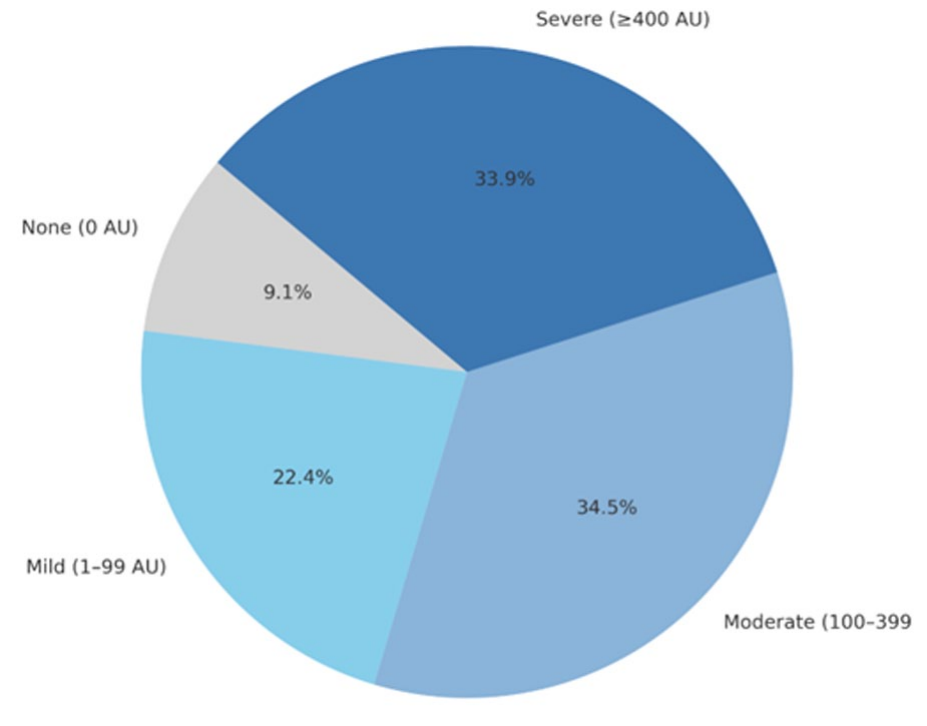
CAC score grade distribution in the full cohort.

The median CAC score was 225 [IQR 41.5–632.5] AU, and the median CAC percentile was 65.0 [IQR 40.5–85.0] AU, both significantly above the MESA reference median (*p* < 0.001; Table 2). Applying commonly used CAC score categories,^21^ only 9% of the patients had a CAC score of 0; 22% had a mild CAC (1–99 AU), 34.5% had moderate CAC (100–399 AU) and 34% had a severe CAC ( ⩾400 AU; Figure 1).

**Table 2.** CAC and CAC percentiles in the overall cohort and across sub-groups.

	CAC score (AU)	*p*-Value	CAC percentile	*p*-Value
Overall cohort	225.0 [41.5–632.5]		65.0 [40.5–85.0]	<0.001
Age				
>65 years (*n* = 127)	226.0 [66.0–686.0]	0.396	61.0 [36.0–80.0]	0.002
⩽65 years (*n* = 38)	197.5 [15.0–423.0]		80.0 [58.2–90.7]	
Sex				
Male (*n* = 78)	159.5 [30.0–479.5]	0.023	65.0 [36.0–82.7]	0.262
Female (*n* = 87)	274.0 [80.0–880.0]		67.0 [41.0–86.0]	

Data shown as median [interquartile range]. *p*-Values represent one sample Wilcoxon signed-rank test comparing CAC percentile to 50th percentile for the overall cohort; Mann–Whitney *U* test for subgroup comparisons.

**Figure 2.**
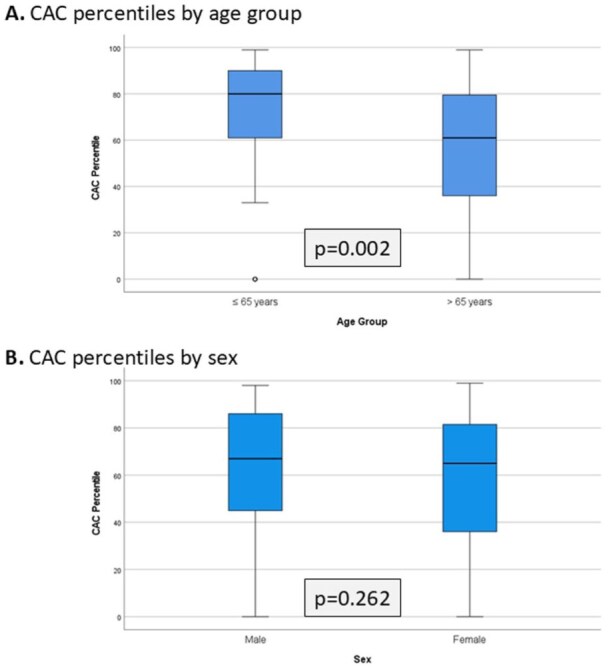
CAC percentiles stratified by age group and sex.

In a subgroup analysis, patients aged 65 years and below had a significantly higher median CAC percentile than patients above 65 years (61.0 [IQR 36.0–80.0] vs 80.0 [58.2–90.7], *p* = 0.002), despite similar absolute CAC scores (*p* = 0.396; Figure 2(a)). While absolute CAC scores differed significantly between sexes (median [IQR]: 159.5 [30.0–479.5] in men vs 274.0 [80.0–880.0] in women, *p* = 0.023), this difference was no longer significant when comparing CAC percentiles (65.0 [36.0–82.7] vs 67.0 [41.0–86.0], *p* = 0.262; Figure 2(b)).

Exploratory analysis of extracoronary atherosclerosis (Table 1) demonstrated a significant stepwise association with CAC burden. In the overall cohort, 84 patients (51%) had aortic arch atherosclerosis and 69 (42%) had carotid artery atherosclerosis. Stratified by CAC category, the prevalence of aortic arch and carotid involvement was 20% and 13% in those with a CAC score of 0 (*n* = 15), 38% and 24% in mild CAC (*n* = 37), 40% and 28% in moderate CAC (*n* = 57), and 78% and 75% in severe CAC (*n* = 56), respectively (*p* < 0.01 for trend).

## Discussion

In this CCTA-based cohort, patients with ESUS demonstrated a high burden of coronary artery calcium, with percentiles exceeding age- and sex-adjusted population norms. Younger patients showed particularly elevated CAC percentiles, suggesting early vascular aging or subclinical atherosclerosis disproportionate to chronological age. These findings suggest that coronary atherosclerosis may contribute to stoke pathogenesis in a subset of ESUS patients and could partly explain the heterogeneity of treatment response observed in anticoagulation trials. Identifying these individuals may enable more tailored prevention strategies.^22–25^

Consistent with these findings, we observed a higher burden of atherosclerosis in the aortic arch and carotid arteries among patients with higher CAC categories, reinforcing the concept of systemic vascular involvement in ESUS.

However, since elevated CAC is also associated with the development of atrial fibrillation,^26,27^ the observed CAC burden may reflect overlapping vascular and arrhythmic risk rather than a purely atherosclerotic mechanism. Moreover, vascular risk factors such as hypertension and diabetes are common to both atrial fibrillation and atherosclerosis, and may underlie the observed association between elevated CAC and ESUS. This overlap complicates mechanistic attribution, as CAC burden may reflect a shared substrate rather than a distinct pathophysiologic pathway. Future studies should explore whether high CAC portends eventual AF detection in this population and how CAC-guided monitoring might be optimized.

The mean age in our cohort was relatively high (73 years old) compared to other ESUS cohorts. This likely reflects local practice patterns, where younger patients are preferentially referred for TEE rather than CCTA. Nonetheless, the prevalence of hypertension and diabetes in our population was consistent with prior studies,^28,29^ supporting the broader relevance of vascular risk profiling in ESUS workup.

Notably, our subgroup analysis revealed that younger patients had significantly elevated CAC percentiles despite similar absolute scores compared to older individuals, suggesting accelerated vascular aging or disproportionate atherosclerotic burden relative to age. Conversely, while women had higher median absolute CAC scores than men, their percentiles—adjusted for age and sex—were similar, highlighting the importance of contextualizing CAC values using population-based reference data. These findings reinforce the utility of percentile-based interpretation, particularly in younger populations where absolute scores may underestimate risk.

This study also emphasizes the value of performing CCTA in patients with ESUS—particularly in younger patients—as it may uncover undiagnosed coronary artery disease, including cases of severe or obstructive atherosclerosis. From a practical standpoint, gated cardiac CT provides mechanistic insights into stroke pathogenesis beyond CAC, including identification of intra-cardiac thrombi and characterization of left atrial morphology. However, even non-gated CT scans—often obtained for other clinical indications—may yield valuable CAC data. This may be especially useful in younger patients, where high CAC percentiles can uncover early atherosclerotic burden not evident through traditional risk factors. CAC scoring could also support therapeutic decisions, such as initiation of statins or prolonged dual antiplatelet therapy, although prospective trials are needed to validate these strategies.^30^ Incorporating CCTA into the diagnostic workup of ESUS could improve risk stratification and guide personalized prevention strategies.

From a research perspective, our findings support prospective studies evaluating CAC as a biomarker for stroke recurrence risk and testing antithrombotic or lipid-lowering strategies stratified by coronary atherosclerotic burden.

## Limitations

This study has several limitations. First, its retrospective design and single-center setting may introduce referral and selection biases. Only patients who underwent CCTA were included, potentially excluding those with different risk profiles or clinical trajectories. Second, we did not include a control group matched for age and sex, nor did we compare CAC burden across other stroke subtypes such as small vessel disease or cardioembolic strokes due to atrial fibrillation. This limits our ability to determine whether CAC elevation is unique to ESUS. Third, while CAC is a validated marker of atherosclerotic burden, it is a surrogate marker and causality cannot be inferred.

Our study did not include systematic long-term cardiac monitoring beyond index hospitalization, and post-discharge AF diagnoses were not uniformly available for analysis. As such, we cannot evaluate whether CAC scores were predictive of subsequent AF detection. This represents a limitation and highlights the need for prospective studies examining whether elevated CAC identifies ESUS patients at increased risk for delayed AF diagnosis or recurrent embolic events.

## Conclusion

Patients with ESUS show a high burden of coronary atherosclerosis, with CAC scores and percentiles exceeding age- and sex-adjusted population norms. These findings support a potential atherosclerotic contribution in a subset of cases and may help explain the limited benefit of empiric anticoagulation in unselected ESUS populations. Given the known association between CAC and future atrial fibrillation, elevated CAC may reflect a broader cardiovascular risk state rather than a purely atherosclerotic mechanism. CAC assessment—readily available via gated or even non-gated CT—may aid in etiologic classification, guide secondary prevention strategies, and identify patients who may benefit from intensified vascular risk modification.
